# Effects of Whole-Body Vibration Training Combined With Cyclic Hypoxia on Bone Mineral Density in Elderly People

**DOI:** 10.3389/fphys.2019.01122

**Published:** 2019-08-30

**Authors:** Marta Camacho-Cardenosa, Alba Camacho-Cardenosa, Martin Burtscher, Javier Brazo-Sayavera, Pablo Tomas-Carus, Guillermo Olcina, Rafael Timón

**Affiliations:** ^1^Faculty of Sport Sciences, University of Extremadura, Cáceres, Spain; ^2^Department of Sport Science, Medical Section, University of Innsbruck, Innsbruck, Austria; ^3^Instituto Superior de Educación Física, Universidad de la República, Rivera, Uruguay; ^4^Polo de Desarrollo Universitario EFISAL, Universidad de la República, Rivera, Uruguay; ^5^Departamento de Desporto e Saúde, Escola de Ciências e Tecnologia, Universidade de Évora, Évora, Portugal; ^6^Comprehensive Health Research Centre (CHRC), Universidade de Évora, Évora, Portugal

**Keywords:** normobaric hypoxia, whole-body vibration, bone mineral density, bone metabolism, osteoporosis

## Abstract

Prevention and treatment of osteoporosis are an issue of great concern in public health so that the increase/maintenance of whole-body bone mineral density (BMD) is clinically relevant and could reduce the financial burden. Whole-body vibration (WBV) has been recently proposed as a potential alternative to bone stimulation, which combined with therapies, could provide a new treatment for osteoporosis prevention. In this sense, moderate cyclic hypoxia protocols may help to restrain osteoclastic activity and/or stimulate osteoblastic activity, enhance the effects of whole-body vibration alone. So, the present study investigated the effects of cyclic hypoxic exposure combined with WBV training on BMD of the elderly. Healthy elderly persons (*n* = 30) were randomly assigned to a (1) Hypoxia-Whole Body Vibration group (HWBV; *n* = 10), (2) Normoxic-Whole Body Vibration group (NWBV; *n* = 10) or (3) Control group (CON; *n* = 10). During 18 weeks, HWBV performed WBV treatment under normobaric hypoxic conditions (16.1% FiO_2_). A vibration session included 4 bouts of 30 s (12.6 Hz–4 mm) with 1 min rest between bouts. NWBV performed the same vibration treatment as HWBV but under normoxic conditions. Whole-body and proximal femur BMD (g⋅cm^−2^) were measured using dual-energy X-ray absorptiometry. Two-way ANOVA indicated a borderline significant (*p* = 0.07) time x group interaction for total BMD; *post hoc* analysis revealed a slight but significant (*p* = 0.021) increase of BMD after treatment in the HWBV group. In conclusion, 18-week WBV training with hypoxic stimuli has shown positive effects for the participants of the current study. As changes did not differ significantly between groups, future large-scale studies will be necessary to confirm these findings.

## Introduction

With increasing life expectancy, the growing proportion of elderly populations across the world has made osteoporosis an increasingly important public health issue (Verschueren et al., 2011). Two hundred million individuals are affected by osteoporosis worldwide (Reginster and Burlet, 2006), causing 8.9 million fractures yearly (1,000 fractures per hour), with the hip injury being the most debilitating (Johnell and Kanis, 2006). This issue is also associated with other health problems such as pain, hospitalisation, surgery, loss of independence and premature death (Harding and Beck, 2017). On this basis, the prevention and treatment of osteoporosis are of utmost importance in public health (Bliuc et al., 2009), implying the need for more research to find ways to reduce the related financial burden (Winklmayr et al., 2015).

Several studies suggested regular physical activity as an effective tool to prevent effects of ageing (Gomez-Cabello et al., 2012; Sallis, 2015) and improve the bone health (Marin-Puyalto et al., 2018). Whole-body vibration (WBV) training may represent an alternative to current methods to fight bone disorders and to reduce the risk of impact injuries (Mikhael et al., 2010). This type of activity, which can be generated with different platforms (vertical, rotational or lateral modes) (McMillan et al., 2017) generates forces that are transferred to the weight-bearing bones of the skeleton as done by other exercise modalities (Cheung and Giangregorio, 2012). So, WBV exercises could have beneficial effects associated with bone formation and the neuromuscular and cognitive functions, reducing the risk of falls and fractures (Moreira-Marconi et al., 2016; Bemben et al., 2018). However, several authors reported conflicting results due to the variety of protocols used (Marin-Puyalto et al., 2018). Some studies have included protocols with frequencies at 10–15 Hz to allow for gentle adaptation in frail populations (elderly or rehabilitation programs, etc.) (Gusi et al., 2006; Turner et al., 2011; von Stengel et al., 2011). These investigations reported a clinically relevant effect in bone loss prevention at the femoral neck and lumbar spine. Although the frequency is important, the duration of WBV intervention plays an important role (Gusi et al., 2006). Authors suggested a dose-response relationship between vibration exposure and reduced bone resorption (Turner et al., 2011). A minimum of three times per week and six months’ duration could be necessary to obtain improvements in bone mass (Gusi et al., 2006; Gomez-Cabello et al., 2012; von Stengel et al., 2012). Activity levels tend to progressively decrease with age; the inactivity is higher among older adults (Schutzer and Graves, 2004). So, a high frequency and duration of training could be inconvenient for the elderly population, which is unable or reluctant to do exercise due to lack of sufficient motivation or interest (Goudarzian et al., 2017; Rosenberg et al., 2018). Variability within the available systems of vibration and lack of consensus on the WBV training protocol result in the conflicting evidence for efficacy on bone health (Fratini et al., 2016; McMillan et al., 2017). The optimal dose of vibration exercise that is needed to increase BMD in humans has yet to be defined (Karakiriou et al., 2011). Other drawbacks of this type of exercise is that benefits have only been found in the hip or lower body (Marin-Puyalto et al., 2018). Therefore, the combination of effects of WBV training with other therapies could provide a new mode of osteoporosis prevention and treatment.

Therapeutic benefits of hypoxic training have been suggested for clinical populations such as the elderly (Millet et al., 2016). Development of exercise in a hypoxic environment could enhance the physiological experience of training (Scott et al., 2014), being a safe and non-invasive strategy for elderly adults (Bayer et al., 2017).

Previous studies have combined cyclic hypoxia and strength training in the elderly population to determine the effects on different parameters, such as muscle strength (Schega et al., 2013; Camacho-Cardenosa et al., 2019a), cognitive performance and postural stability (Stadelmann et al., 2015). Regarding bone parameters, two previous studies applied hypoxia training in healthy active or trained subjects; they showed increased or maintained bone mineral density (BMD) after 8 and 7 weeks, respectively (Ramos Campo et al., 2015; Martinez-Guardado et al., 2019). To the best of our knowledge, the combined effects of WBV training and hypoxic exposure on health parameters have not been investigated. Additional studies are needed to determine the optimal cyclical dose that could have beneficial effects on bone systems.

In the bone microenvironment, which is physiologically hypoxic, various genes downstream of HIF mediate different effects (Dirckx et al., 2018). Hypoxia-regulated transcription activates genes and pathways that reduce oxygen consumption and the cellular dependence on oxygen (Semenza, 2012). On the other hand, HIF-regulated genes induce angiogenesis and erythropoiesis to increase the tissue oxygen supply; it even stimulates bone formation (Dirckx et al., 2018). However, different modes of hypoxic exposure may lead to different impacts on bone metabolism (Camacho-Cardenosa et al., 2019b). While sustained (Baladia et al., 2013; Basu et al., 2014; Rittweger et al., 2016; O’Brien et al., 2018) and intermittent hypoxia (Tomiyama et al., 2008; Terzi and Yilmaz, 2016) related to obstructive sleep apnoea might inhibit osteogenic differentiation and promote osteoclast function, cyclical hypoxia has been presented as a promising strategy to beneficially impact bone metabolism (Guner et al., 2013; Wang et al., 2016). In this way, the combining of WBV training and cyclic hypoxic exposure could augment the beneficial effects of WBV training, resolve the inconvenience of long training periods, and ensure that the bone benefits aren’t just localised.

Considering all these findings, the present study investigated the effects of normobaric cyclic hypoxic exposure combined with WBV training on BMD of the elderly (age > 65 years) over an 18-week period. We hypothesised that WBV training with hypoxic stimuli would maintain or increase BMD compared to WBV training or resting in normoxia.

## Materials and Methods

### Study Design

This was a randomised double-blind controlled study. There were separate intervention and assessment teams. We tried to blind the study for participants, as they were trained/tested separately. During 18 weeks the volunteers completed 36 training sessions supervised by an experienced member of the research group. The frequency of training was twice a week; sessions were scheduled with at least one day of rest in between for optimal recovery. All patients were assessed at two time points by a group of researchers, who were blinded to the treatment assignment. Outcomes were measured at baseline (Pre) before the 18 weeks of intervention and reassessed 7 days after the last session (Post). Participants were instructed to abstain from participating in any other type of physical exercise during the duration of this trial.

### Participants

The voluntary participation of elderly subjects was requested by letter and verbal communication at the Senior Universities and pensioners’ associations of Évora (Portugal). Inclusion criteria, assessed during a screening visit, were: (1) women and men aged 65 years or older, (2) absence of participation in any other type of intervention based on physical exercise in the last 6 months in order to avoid interactions with the previous practice, (3) subjects have not been above 1500 m during the last 3 months, (4) no current medical condition not compatible with planned exercise, (5) free of illness or medication potentially affecting the bone system, (6) estimated daily calcium intake of 500 mg/day or more and (7) consumption of no more than four alcoholic beverages per week. Exclusion criteria were mainly based on contra indications for WBV (severe cardiovascular diseases, ocular diseases that affect the retina, neuromuscular and heart diseases, stroke, implant, bypass, stent, arthritis and other joint disease or epilepsy) or a frequency of participation in the stipulated program lower 80% (participants who missed more than 20% of training sessions were excluded).

**FIGURE 1:**
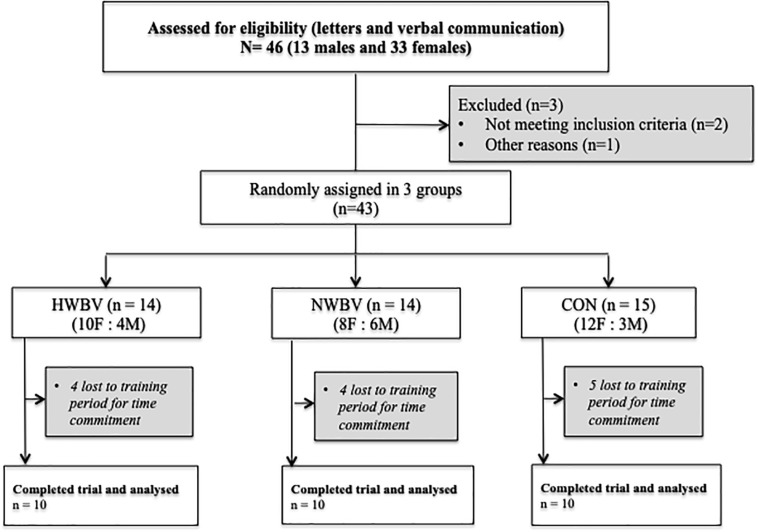
Flow of participants through each stage of the trial. HWBV, hypoxia whole body vibration group. NWBV, normoxia whole body vibration group; CON, control group; F, female; M, male.

Forty-six volunteers (13 males and 33 females) were informed about the study procedures and were requested to sign a declaration that they voluntarily consented to participate in this research. The eligible volunteers, who met the inclusion criteria (*n* = 43; age: 72 ± 5.3 years; BMI: 29 ± 4.2 kg⋅m-2;% fat: 36.5 ± 8.2; BMD: 1.069 ± 0.115 g⋅cm-2), were randomly divided into three groups, by simple randomisation: (1) Hypoxia Whole Body Vibration group (HWBV; *n* = 14), who performed whole body vibration treatment under normobaric hypoxic conditions; (2) Normoxic Whole Body Vibration group (NWBV; *n* = 14), who performed whole body vibration treatment under normoxic conditions; and (3) Control group (CON; *n* = 15), who were instructed to continue with their normal daily activities for the entire duration of the study (Figure 1).

All procedures were performed in studies involving human participants were in accordance with the 1964 Helsinki declaration and its later amendments or comparable ethical standards and the study design was approved by the Bioethical and Biosecurity Commission of the University of Extremadura (17/2016).

### Training Protocol

During sessions, HWBV and NWBV groups performed the vibration exercise in a standing position, with feet side by side on specific landmark in the board and barefoot to eliminate any damping of the vibration caused by footwear (Gusi et al., 2006). The angle of flexion of the knee during the vibration exercise was set at 120°, which were established with goniometer. The participants were allowed to hold the vibration platform with their hands ant the intervention was supervised by an experienced member of the research group for correct postural control. Vibration stimulus (12.6 Hz) was produced and provided by a sinusoidal vibration platform (Galileo 2000, Novotec GmbH, Pforzheim, Germany) and the distant position form the axis of rotation was 4 mm peak-to-peak. The effective acceleration (a; 2.55 g force) was calculated using frequency (f in hertz) and displacement (d in metres) provided by the manufacturer according to the equation: *a* = (2π*f*)^2^*d* (Griffin, 1997). Units of this equation are g that represents the Earth’s gravitational acceleration at 9.81 m/s^2^. Subjects performed four sets of 30 s of vibration per session, separated by 60 s of rest. The total duration of the training session was about 16 min, which included a 10-min warm-up with 5 min bicycling at 25–50 W and 40–50 rpm and another 5 min of stretching exercises. All of this took place in a hypoxia chamber (CAT 310, Lousiville, Colorado, United States) placed in the laboratory.

For the HWBV group, fraction of inspired oxygen (FiO_2_) was set to 16.1% (0.16) to simulate an altitude of 2500 m above sea level; FiO2 was controlled regularly with an electronic device (HANDI+, Maxtec, Salt Lake City, Utah, United States). In order to blind subjects to altitude, the system was also run for the NWBV group with normoxic airflow into the chamber (up to 1000 l/min) and produced the same audible noise as in the hypoxic condition. NWBV subjects inspired FiO_2_ of 21.0% (0.21) to simulate an altitude of 459 m above sea level. Furthermore, all systems were covered with fabric to prevent participants from visually identifying the normoxic or hypoxic conditions.

At each session, peripheral oxygen saturation (SpO_2_) was controlled using a finger pulse-oximeter (Konica Minolta, Japan) and heart rate (HR) using a heart rate monitor (Polar team 2, Polar, Finland) to know the physiological challenge posed on the participants in different times of the session. Above parameters were controlled during the second minute of the warm-up (warm-up), previously to the start of the whole-body vibration protocol (pre-training), between the second and the third set of the whole body vibration protocol (mid-training) and after the whole body vibration protocol (post-training).

### Measurements

#### Socio-Demographic Data and Lifestyle Questionnaires

A general questionnaire was administered to collect medical and demographic data to check the inclusion/exclusion criteria. As control variables, prior and after the intervention, calcium intake was estimated using a food frequency questionnaire adapted to Portuguese traditional foods and dishes (Ribeiro et al., 2006). The bone-specific physical activity questionnaire (B-PAQ) was used to assess the physical activity level of the participants in the last 12 months. Respondents recorded type, frequency and years of physical activity involvement to assess the effects of mechanical loading on the skeleton (Weeks and Beck, 2008).

#### Body Composition

Body composition variables such as percentage fat mass (CV = 2.1%) and percentage lean mass (CV = 1.7%) were obtained using dual-energy X-ray absorptiometry (DXA, Norland Excell Plus; Norland Inc., Fort Atkinson, United States). The same experienced technician performed all the scans, which were analysed by a graphical user interface (GUI) to Windows XP operating system. Body mass index (BMI) was determined per the accepted method (BMI = weight/height^2^, kg⋅m^–2^).

#### BMD Assessment

BMD values from the whole body and right proximal femur (femur total, trochanter and intertrochanteric region) were assessed using DXA (CV = 1.9%). The same experienced technician performed all the scans, which were analysed by a graphical user interface (GUI) to Windows XP operating system. BMD was expressed in g⋅cm^–2^ and as the difference, expressed as standard deviations (SD), from the normal average value of peak bone mass at a participant’s age. *T*-scores were calculated using the National Health and Nutrition Examination Study (NHANES) III BMD norms, as recommended by the World Health Organization for the diagnosis of osteoporosis in clinical practice (Looker et al., 2012).

#### Compliance Training

Compliance training was calculated as the number of sessions completed divided by the 36 possible sessions available per participant.

### Statistical Analysis

Statistical analyses were performed using the statistical analysis package SPSS v.20 for MAC (IBM, New York, United States). Standard statistical methods were used for the calculation of descriptive statistics as the mean and 95% Confidence Intervals. Kolmogorov–Smirnov tests were conducted to show the distribution of the studied variables and Levene’s test for homogeneity of variance.

One-way analysis of variance (ANOVA) was used to compare differences across groups at baseline. Analysis of chi-square was also applied to evaluate differences between groups in the initial data on sex. Two-way repeated measures ANOVA with Bonferroni *post hoc* tests were used to investigate the main effects and the interaction between group-factor (control vs. normoxia vs. hypoxia) and time-factor (pre-training vs. post-training), considering the sex as covariate. The effect size (Cohen, 1992) was calculated for all variables between baseline and after 18 weeks of intervention. The magnitude of change considered was trivial (<0.5), small (0.5–1.25), moderate (1.26–1.99) or large (>2.00) (Rhea, 2004). Additionally, absolute change and the percentage change from pre- to post-test was calculated for all variables for each group, and the effects of the interventions were evaluated by pre-specifying the minimum detectable change (MDC). The MDC is an absolute measure of reliability (measurement error), which accounts for various sources of variability in defining a confidence interval in units of the measure. These values are being increasingly used to assist in interpreting results and determining whether a change between repeated tests is random variation or a true change in performance (Haley and Fragala-Pinkham, 2006). An effect was considered relevant when the change was greater than MDC. The *p* < 0.05 criterion was used for establishing statistical significance.

## Results

A total of 30 elderly individuals completed the training program and all of the assessments and were subsequently included in the analysis. During the training period, thirteen subjects dropped out because of prolonged illnesses or time commitment (Figure 1) and were excluded from analysis. None of the dropouts left the program as a result of injury or adverse responses to the training. The compliance with training prescription was 100% in the NWBV group and 91.39% in the HWVB group.

The characteristics of the final subject at baseline are represented in Table 1. Prior to the training, no significant differences between groups were observed for any variable.

**TABLE 1:** Baseline characteristics of the sample.

	**HWBV *N* = 10**	**NWBV *N* = 10**	**CON *N* = 10**	***P***
Age (years)	73.50(70.11−76.89)	69.00(65.21−72.79)	73.40(69.81−−76.99)	0.091
BMI (kg⋅m^–2^)	28.86(25.85−31.86)	29.38(25.77−32.99)	28.86(26.35−31.36)	0.951
Fat mass (%)	37.02(30.83−43.22)	34.11(27.37−40.86)	38.26(33.49−43.03)	0.529
Lean mass (%)	62.98(56.78−69.17)	62.95(52.29−73.61)	61.74(56.97−66.50)	0.957
Past BPAQ (score)	8.03(3.69−12.38)	10.17(4.23−16.11)	6.92(1.28−12.56)	0.619
Total BMD (gr.cm^–2^)	1.061(0.977−1.145)	1.112(1.018−1.21)	1.034(0.968−1.100)	0.321
Sex:				0.155
- Male^a^	3(30.00%)	5(50.00%)	2(20.00%)	
- Female^a^	7(70.00%)	5(50.00%)	8(80.00%)	

*Values expressed as Mean (95% Confidence Intervals). *P* values of analysis of variance (ANOVA). ^*a*^values expressed as N (%). *P* values of analysis of Chi-square.*

No significant differences were found for estimated calcium intake (*p* > 0.05) in all groups between PRE (NWBV: 921.27 ± 419.30 mg/day; HWBV: 1124.30 ± 421.52 mg/day nor CON: 928.80 ± 291.89 mg/day) and POST (NWBV: 848.00 ± 229.81 mg/day; HWBV: 1186.80 ± 468.97 mg/day nor CON: 969.80 ± 392.73 mg/day) intervention.

Because of the neutral smell of the conditioned air, subjects could not tell if they were in hypoxia or normoxia. Blinding was successful as more than 60% of subjects guessed their group incorrectly.

**FIGURE 2:**
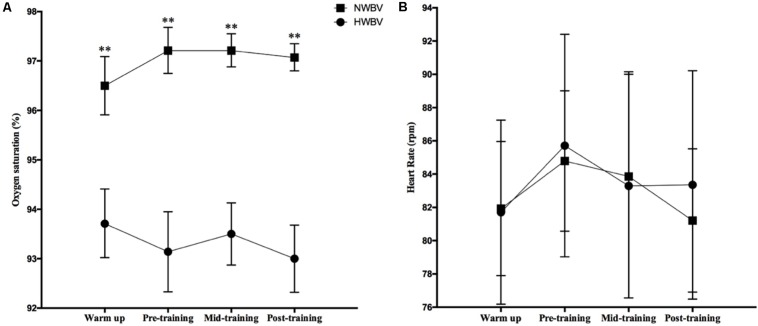
Physiological challenges of oxygen saturation **(A)** and heart rate **(B)** controlled in the second minute of warm-up (warm up), before whole body vibration protocol (pre-training), between second and third set of whole body vibration protocol (mid-training) and after whole body vibration protocol (post-training). Significantly differences from HWBV: ^∗∗^*p* < 0.01. Values are means and 95% Confidence Intervals. NWBV, normoxia whole body vibration group; HWBV, hypoxia whole body vibration group.”

The oxygen saturation was lower (*p* < 0.01) for the hypoxia groups in all measured points during the training session. HR during the training sessions was not significantly different between groups (Figure 2).

Results of bone parameter responses after 18 weeks of intervention are shown in Table 2. Because of the responses to the training showed no sex differences, results for male and female participants were combined and analysed together. Changes in total and proximal femur BMD were not significantly different between groups. Two-way ANOVA indicated a borderline significant (*p* = 0.072) time x group interaction for total BMD and *post hoc* analysis revealed a slight but significant (*p* = 0.021) increase of this parameter after treatment in the HWBV group.

**TABLE 2:** Bone Mineral Density measurement outcomes (g⋅cm^–2^) at baseline and after 18 weeks of normoxia-whole body vibration exercise (*N* = 10), combined hypoxia-whole body vibration exercise (*N* = 10) and in control group (*N* = 10).

	**Baseline**	**Δ (%)**	**18 weeks**	**ES**	**SP**	**MDC (%)**	**ANOVA (p)**		
							
							**Time**	**Group**	**Time x Group**
**Total**									
NWBV	1.112 (1.018–1.206)	–1.17	1.099 (1.005–1.193)	0.08	52.0	4.66	0.421	0.424	0.072
HWBV	1.061 (0.977–1.145)	+ 3.58	1.099 (1.021–1.177)	0.35		6.75			
CON	1.034 (0.968–1.100)	+ 0.68	1.041 (0.973–1.109)	0.11		2.38			
**Total T-score**									
NWBV	−0.967 (−2.379–0.446)	+ 20.68	−1.167 (−3.175–0.841)	0.29	64.1	39.19	0.449	0.696	1.000
HWBV	−1.257 (−1.653–0.862)	–48.85	−0.643 (−1.099–0.187)	1.35		77.19			
CON	−0.950 (−1.861–0.039)	–10.53	−0.850 (−1.804–0.104)	0.09		32.59			
**Femoral**									
NWBV	0.889 (0.792–0.986)	+ 1.57	0.903 (0.809–0.997)	0.07	14.2	3.46	0.913	0.560	0.543
HWBV	0.837 (0.716–0.958)	+ 1.19	0.847 (0.726–0.968)	0.06		1.76			
CON	0.830 (0.728–0.932)	0.96	0.838 (0.744–0.932)	0.07		6.91			
**Femoral T-score**									
NWBV	−0.744 (−1.383–0.106)	–13.44	−0.644 (−1.232–0.058)	0.13	10.4	26.63	0.877	0.658	0.684
HWBV	−1.070 (−1.840–0.300)	–3.74	−1.030 (−1.804–0.256)	0.04		15.60			
CON	−1.040 (−1.801–0.279)	–6.73	−0.970 (−1.697–0.243)	0.07		45.29			
**Trochanter**									
NWBV	0.697 (0.600–0.793)	+ 1.00	0.704 (0.610–0.799)	0.00	65.5	4.67	0.126	0.527	1.000
HWBV	0.660 (0.556–0.764)	+ 0.61	0.664 (0.560–0.768)	0.00		1.92			
CON	0.651 (0.570–0.732)	–2.00	0.638 (0.560–0.716)	0.09		5.85			
**Intertrochanteric**									
NWBV	1.048 (0.937–1.159)	+ 2.48	1.074 (0.964–1.185)	0.14	57.0	3.76	0.798	0.369	0.670
HWBV	0.977 (0.846–1.108)	+ 1.43	0.991 (0.857–1.125)	0.05		2.33			
CON	0.960 (0.845–1.075)	+ 1.56	0.975 (0.872–1.078)	0.07		8.92			

*Values expressed as Mean (95% Confidence Intervals). NWVB, normoxia whole body vibration group; HWBV, hypoxia whole body vibration group; CON, control group; Δ, absolute change; ES, effect size; SP, statistical power; MDC, minimum detectable change; *p*, *p* values.*

**FIGURE 3:**
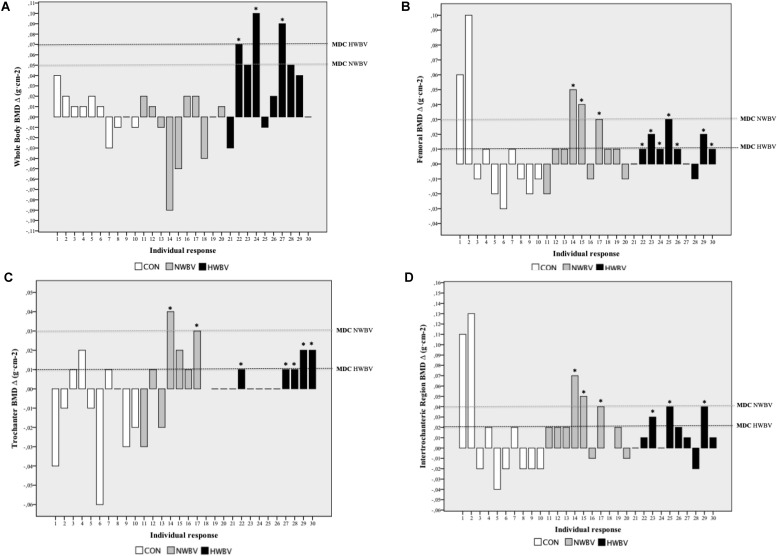
Heterogeneity of whole-body BMD **(A)**, femoral *T*-score **(B)**, trochanter BMD **(C)** and intertrochanteric region BMD **(D)** values changes absolute, following whole body vibration alone (NWBV) and combined with normobaric hypoxia (HWBV). Dark dash line: minimal detectable change (MDC) to HWBV group; light dash line: minimal detectable change to NWBV group; ^∗^: intra-individual difference equal or greater than MDC (i.e., 0.07 g⋅cm^– 2^ of whole body BMD); Δ: absolute change (post- minus pre-training absolute change).

Figure 3 shows the whole body and right proximal femur BMD individual responses after 18 weeks of intervention. Based on MDC, 30% of the subjects of HWBV group (3/10) reached this value in whole body BMD (MDC = 0.07 g⋅cm^–2^). In the femoral BMD, 70% (7/10) of the subjects reached the MDC following HWBV (MDC = 0.01 g⋅cm^–2^) and 30% (3/10) following NWBV (MDC = 0.03 g⋅cm^–2^). Fifty percent of the subjects (5/10) showed trochanter BMD changes equal of MDC after HWBV (MDC = 0.01 g⋅cm^–2^) and 20% (2/10) in the NWBV group. Thirty percent (3/10) of the subjects reached the MDC in the intertrochanteric region BMD following HWBV (MDC = 0.02 g⋅cm^–2^) and NWBV (MDC = 0.04 g⋅cm^–2^).

## Discussion

To the best of our knowledge, this is the first study evaluating the effects of prolonged (18 weeks) WBV training combined with normobaric cyclic hypoxic exposure on BMD in elderly people. WBV training under normobaric cyclic hypoxia tended to increase total BMD. Furthermore, the results indicate that the hypoxic stimulus combined with WBV training may cause an individualised response demonstrating effects in the proximal femur BMD after 18 weeks of treatment.

Our findings are similar to those reported by Beck and Norling (2010). These researchers achieved the maintenance of bone density at the proximal femur after two sessions per week of low-frequency vibratory training and found a significant loss of trochanteric cortical density in their control group. Although BMD maintenance can be considered positive due to the fact that elderly adults face rapid bone loss (Karakiriou et al., 2011), three times per week of WBV training could be necessary to obtain improvements in the hip region (Gusi et al., 2006). Therefore, an higher amount of vibration exposure compared with that performed in the present study may cause larger effects (Gomez-Cabello et al., 2012). The combination of WBV training with physical activity and other therapies could decrease the participation for a population which is unable or unwilling to significantly increase their level of physical activity (Rosenberg et al., 2018).

Based on the results of the present study, the WBV combined with normobaric cyclic hypoxia treatment tends to improve total BMD and to cause a greater individualised response regarding changes in the proximal femur BMD. In this context, subjects with BMD gains had a lower risk of fracture and those with BMD losses had a higher risk (Austin et al., 2012). Thus, 2–6% change in BMD is associated with a 15–40% decrease in fracture risk (Bouxsein et al., 2019).

Although hip fracture is considered as most debilitating, profound loss of skeletal mass results in bone fragility and susceptibility to fractures at the spine, wrist and forearm as a result of falling (Harding and Beck, 2017). Thus, the increase/maintenance of whole body BMD is clinically relevant and could reduce the financial burden for public health. We hypothesised that the changes in total BMD observed before that of more specific regions, might be due to the effect of hypoxia, which was administered in a general manner being the subjects trained by breathing oxygen-depleted air. Unfortunately, there is limited research regarding the effect of cyclic hypoxic training on bone metabolism.

Previous authors have suggested that hypoxic training could enhance the physiological experience of training (Scott et al., 2014). Hypoxic stimulus and WBV training could have synergistic effects on bone metabolism. Only two studies previously have investigated the effects of hypoxic training on BMD. As in the results of this investigations healthy active adults showed improved total BMD after 8 weeks of normobaric hypoxic training (Martinez-Guardado et al., 2019). However, in trained triathletes, 7 weeks of normobaric cyclic hypoxia training at 15% PiO_2_ (2 days per week; 60 min per session) resulted in no reported changes in total BMD (Ramos Campo et al., 2015). In this sense, hypoxic protocols such as the one applied to the population that participated in the study could explain these different responses.

Based on *in vitro* studies, various mechanisms could explain the effects of hypoxia exposure on bone metabolism. Firstly, HIF-mediated upregulation of such glycolytic enzymes as pyruvate dehydrogenase kinase 1 (PDK1), lactate dehydrogenase A (LDHA) and glucose transporters (GLUTs) compensate for the energy inefficiency of glycolysis (Semenza, 2001), since aerobic glycolysis is the main metabolic pattern during osteoblastic differentiation (Lee et al., 2017). Thus, hypoxia exposure might enhance bone formation by promoting glycolysis as the main metabolic pathway. However, high bone mass does not depend solely on glycolysis (Dirckx et al., 2018). HIF also regulates bone remodelling-involved genes such as vascular endothelial growth factor (VEGF), erythropoietin (EPO) and osteoprotegerin (OPG), the factor that inhibits osteoclastogenesis by counteracting RANKL and therefore bone reabsorption (Wu et al., 2015). VEGF represents a key player in the coupling of angiogenesis and osteogenesis (Dirckx et al., 2018) and in promoting osteogenesis (Fuller et al., 2006; Guner et al., 2013). EPO has also been shown to stimulate bone formation and repair (Wu et al., 2014).

On the other hand, some studies demonstrated that hypoxic exercise, similarly to exercise performed in normoxia, augments oxidative stress (Debevec et al., 2017). The effects of oxidative stress caused by WBV training in hypoxic environment on bone metabolism remains to be investigated; however, moderate cyclic hypoxia protocols may reinforce the immune system, while suppressing the production of pro-inflammatory mediators in the bone systems (Serebrovskaya et al., 2011). Rats exposed to cyclic hypoxia (10 min, 13% O_2_, 10-min intervals, 4 h/day, 28 days) had increased alkaline phosphatase activity (Litovka, 2008) due to high osteoblast activity increasing new bone formation. Moreover, rats exposed to hypobaric cyclic hypoxia (430 mmHg, 34 mmHg PaO_2_; 5 h/day, 5 days/week, 5 weeks) showed higher BMD levels (Guner et al., 2013). These effects in rats may result from increased nitric oxide levels (Navarrete-Opazo and Mitchell, 2014). Increased reactive oxygen species production will activate pro-inflammatory cytokines, which cause production of NO in osteoblasts and osteoclasts, among other cells. It is known that NO regulates osteoclast-mediated bone resorption, activates osteoblastic activity and inhibits RANKL expression (Fan et al., 2006; Wang et al., 2008). As a result of all these mechanisms, moderate cyclic hypoxia protocols may inhibit osteoclastic activity and/or stimulate osteoblastic activity; nevertheless, more research is needed to understand these mechanisms (Navarrete-Opazo and Mitchell, 2014).

Several limitations deserve comment. Most important, the sample size of the present investigation is relatively small; this might explain why we were not able to detect significant changes between the groups. The calculation of sample size *a priori* could be crucial in clinical studies to be able to generalise the obtained result to the overall population (Kadam and Bhalerao, 2010). Unfortunately, our sample size was limited due to logistic conditions (availability of rooms, devices, etc.). Thus, power analysis was performed *a posteriori*, which permits knowing the likelihood of having significant effect, given the observed effect size, sample size and predetermined critical significance level. Due to difficulties in getting participants from this population, who have problems with their mobility or do not like physical activity (Bolam et al., 2013), both men and women were included in this study, resulting in different numbers of males and females. Chi-square tests showed that the groups were homogeneous at baseline regarding sex. Furthermore, as no sex by training interactions were found, the results for male and female participants were combined and analysed together. Additionally, we did not measure the serum and urinary levels of bone metabolism markers, which could offer more information about the effect of normobaric cyclic hypoxic exposure and WBV training on bone metabolism. Assessment of molecular signaling pathways during cyclic hypoxia exposure could also help to elucidate mechanisms responsible for adaptations. On the other hand, although DXA is the most widely used bone densitometry technique among the reported studies, the ability of peripheral quantitative computed tomography (pQCT) to assess bone geometric properties may prove advantageous in evaluating the effects of treatment on bone health (Gomez-Cabello et al., 2012). However, the sensitivity of those instruments in detecting subtle exercise-induced changes in bone geometry has not been exhaustively tested. Bone geometry could be monitored by DXA-derived BMD and this technique could provide appropriate information to design optimal therapeutic exercise programmes (Harding and Beck, 2017). Finally, results were obtained in the context of an 18-week trial, and more long-term clinical effects remain unknown. Research on the effects of the training concluded that regarding bone mass among elderly people, continued exercise training is needed to maintain bone mass (Gomez-Cabello et al., 2012).

## Conclusion

An 18-week WBV training with hypoxic stimuli tended to generate positive effects on BMD in the elderly population. Although changes did not differ significantly between groups, the benefits observed within the WBV plus hypoxia group may be promising, but need to be confirmed in future large-scale studies. WBV training generates shear stresses on the bone cells, and this might be enhanced by effects related to the hypoxic stimuli. Future research will provide a deeper understanding of mechanisms responsible for the effects of normobaric hypoxia combined with WBV training.

## Data Availability

The datasets generated for this study are available on request to the corresponding author.

## Ethics Statement

All procedures performed in studies involving human participants were in accordance with the Declaration of Helsinki (1964) and its later amendments or comparable ethical standards and the study design was approved by the Bioethical and Biosecurity Commission of the University of Extremadura (17/2016).

## Author Contributions

MC-C designed the research study, conducted the experiments, and wrote the manuscript. AC-C acquired and analysed the data, and wrote the manuscript. MB analysed the data, and wrote and proofread the manuscript. JB-S and RT designed the research study and wrote the final version of the manuscript. PT-C conducted the experiments, provided the material, and acquired the data. GO conducted the experiments and provided the material.

## Conflict of Interest Statement

The authors declare that the research was conducted in the absence of any commercial or financial relationships that could be construed as a potential conflict of interest.

## References

[B1] Austin M. Yang Y. C. Vittinghoff E. Adami S. Boonen S. Bauer D. C. (2012). Relationship between bone mineral density changes with denosumab treatment and risk reduction for vertebral and nonvertebral fractures. *J. Bone. Miner. Res.* 27 687–693. 10.1002/jbmr.1472 22095631 PMC3415619

[B2] Baladia E. Basulto J. Manera M. (2013). Overestimation of the prevalence of risk of inadequate calcium intake in spanish schoolchildren? Comparison of observable intake with dietary reference intakes; use of the estimated average requirement (ear) versus the recommended dietary allowances (RDA)]. *Nutr Hosp.* 28 971–972. 10.3305/nh.2013.28.3.6313 23848129

[B3] Basu M. Malhotra A. S. Pal K. Kumar R. Bajaj R. Verma S. K. (2014). Alterations in different indices of skeletal health after prolonged residency at high altitude. *High Alt. Med. Biol.* 15 170–175. 10.1089/ham.2013.1098 24666002

[B4] Bayer U. Likar R. Pinter G. Stettner H. Demschar S. Trummer B. (2017). Intermittent hypoxic-hyperoxic training on cognitive performance in geriatric patients. *Alzheimers Dement.* 3 114–122. 10.1016/j.trci.2017.01.002 29067323 PMC5651371

[B5] Beck B. R. Norling T. L. (2010). The effect of 8 mos of twice-weekly low- or higher intensity whole body vibration on risk factors for postmenopausal hip fracture. *Am. J. Phys. Med. Rehabil.* 89 997–1009. 10.1097/PHM.0b013e3181f71063 21403595

[B6] Bemben D. Stark C. Taiar R. Bernardo-Filho M. (2018). Relevance of whole-body vibration exercises on muscle strength/power and bone of elderly individuals. *Dose Response* 16:1559325818813066. 10.1177/1559325818813066 30559636 PMC6291875

[B7] Bliuc D. Nguyen N. D. Milch V. E. Nguyen T. V. Eisman J. A. Center J. R. (2009). Mortality risk associated with low-trauma osteoporotic fracture and subsequent fracture in men and women. *JAMA* 301 513–521. 10.1001/jama.2009.50 19190316

[B8] Bolam K. A. van Uffelen J. G. Taaffe D. R. (2013). The effect of physical exercise on bone density in middle-aged and older men: a systematic review. *Osteoporos Int.* 24 2749–2762. 10.1007/s00198-013-2346-2341 23552825

[B9] Bouxsein M. L. Eastell R. Lui L. Y. Wu L. A. de Papp A. E. Grauer A. (2019). Change in bone density and reduction in fracture risk: a meta-regression of published trials. *J. Bone. Miner. Res.* 34 632–642. 10.1002/jbmr.3641 30674078

[B10] Camacho-Cardenosa M. Camacho-Cardenosa A. Brazo-Sayavera J. Olcina G. Tomas-Carus P. Timon R. (2019a). Evaluation of 18-week whole-body vibration training in normobaric hypoxia on lower extremity muscle strength in an elderly population. *High Alt. Med. Bio.l* 20 157–164. 10.1089/ham.2018.0129 31021265

[B11] Camacho-Cardenosa M. Camacho-Cardenosa A. Timon R. Olcina G. Tomas-Carus P. Brazo-Sayavera J. (2019b). Can hypoxic conditioning improve bone metabolism? a systematic review. *Int. J. Environ. Res. Public Health* 16:E1799. 10.3390/ijerph16101799 31117194 PMC6572511

[B12] Cheung A. M. Giangregorio L. (2012). Mechanical stimuli and bone health: what is the evidence? *Curr. Opin. Rheumatol.* 24 561–566. 10.1097/BOR.0b013e3283570238 22832826

[B13] Cohen J. (1992). A power primer. *Psychol. Bull.* 112 155–159. 19565683 10.1037//0033-2909.112.1.155

[B14] Debevec T. Millet G. P. Pialoux V. (2017). Hypoxia-induced oxidative stress modulation with physical activity. *Front. Physiol.* 8:84. 10.3389/fphys.2017.00084 28243207 PMC5303750

[B15] Dirckx N. Tower R. J. Mercken E. M. Vangoitsenhoven R. Moreau-Triby C. Breugelmans T. (2018). Vhl deletion in osteoblasts boosts cellular glycolysis and improves global glucose metabolism. *J. Clin. Invest.* 128 1087–1105. 10.1172/JCI97794 29431735 PMC5824856

[B16] Fan X. Rahnert J. A. Murphy T. C. Nanes M. S. Greenfield E. M. Rubin J. (2006). Response to mechanical strain in an immortalized pre-osteoblast cell is dependent on ERK1/2. *J. Cell Physiol.* 207 454–460. 10.1002/jcp.20581 16419041

[B17] Fratini A. Bonci T. Bull A. M. (2016). Whole body vibration treatments in postmenopausal women can improve bone mineral density: results of a stimulus focussed meta-analysis. *PLoS One* 11:e0166774. 10.1371/journal.pone.0166774 27907000 PMC5132247

[B18] Fuller D. D. Golder F. J. Olson E. B. Jr. Mitchell G. S. (2006). Recovery of phrenic activity and ventilation after cervical spinal hemisection in rats. *J. Appl. Physiol.* 100 800–806. 10.1152/japplphysiol.00960.2005 16269524

[B19] Gomez-Cabello A. Ara I. Gonzalez-Aguero A. Casajus J. A. Vicente-Rodriguez G. (2012). Effects of training on bone mass in older adults: a systematic review. *Sports Med.* 42 301–325. 10.2165/11597670-000000000-00000 22376192

[B20] Goudarzian M. Ghavi S. Shariat A. Shirvani H. Rahimi M. (2017). Effects of whole body vibration training and mental training on mobility, neuromuscular performance, and muscle strength in older men. *J. Exerc. Rehabil.* 13 573–580. 10.12965/jer.1735024.512 29114533 PMC5667605

[B21] Griffin M. J. (1997). *Vibration and Motion. In Handbook of Human Factors and Vibration.* New York, NY: John Wiley and Sons.

[B22] Guner I. Uzun D. D. Yaman M. O. Genc H. Gelisgen R. Korkmaz G. G. (2013). *The Effect of Chronic Long-term Intermittent Hypobaric Hypoxia on Bone Mineral Density in rats: Role of Nitric oxide.* Totowa: Humana Press. 10.1007/s12011-013-9722-8 23771686

[B23] Gusi N. Raimundo A. Leal A. (2006). Low-frequency vibratory exercise reduces the risk of bone fracture more than walking: a randomized controlled trial. *BMC Musculoskelet. Disord.* 7:92. 17137514 10.1186/1471-2474-7-92 PMC1693558

[B24] Haley S. M. Fragala-Pinkham M. A. (2006). Interpreting change scores of tests and measures used in physical therapy. *Phys. Ther.* 86 735–743. 16649896

[B25] Harding A. T. Beck B. R. (2017). Exercise, osteoporosis, and bone geometry. *Sports* 5:29. 10.3390/sports5020029 29910388 PMC5968984

[B26] Johnell O. Kanis J. A. (2006). An estimate of the worldwide prevalence and disability associated with osteoporotic fractures. *Osteoporos. Int.* 17 1726–1733. 10.1007/s00198-006-0172174 16983459

[B27] Kadam P. Bhalerao S. (2010). Sample size calculation. *Int. J. Ayurveda. Res.* 1 55–57. 10.4103/0974-7788.59946 20532100 PMC2876926

[B28] Karakiriou S. K. Douda H. T. Smilios I. G. Volakis K. A. Tokmakidis S. (2011). Effects of vibration and exercise training on bone mineral density and muscle strength in postmenopausal women. *Eur. J. Sport Sci.* 12 81–88. 10.1080/17461391.2010.536581

[B29] Lee W. C. Guntur A. R. Long F. Rosen C. J. (2017). Energy metabolism of the osteoblast: implications for osteoporosis. *Endocr. Rev.* 38 255–266. 10.1210/er.20172064 28472361 PMC5460680

[B30] Litovka I. H. (2008). Alimentary and oxygen deprivation as the modulator of the bone tissue physiological remodelling rate in young rats. *Fiziol. Zh.* 54 85–93. 18416190

[B31] Looker A. C. Melton L. J. III Borrud L. G. Shepherd J. A. (2012). Lumbar spine bone mineral density in US adults: demographic patterns and relationship with femur neck skeletal status. *Osteoporos. Int.* 23 1351–1360. 10.1007/s00198-011-1693-z 21720893

[B32] Marin-Puyalto J. Gomez-Cabello A. Gonzalez-Aguero A. Gomez-Bruton A. Matute-Llorente A. Casajus J. A. (2018). Is vibration training good for your bones? an overview of systematic reviews. *Biomed Res. Int.* 2018:5178284. 10.1155/2018/5178284 30519579 PMC6241242

[B33] Martinez-Guardado I. Ramos-Campo D. J. Olcina G. J. Rubio-Arias J. A. Chung L. H. Marin-Cascales E. (2019). Effects of high-intensity resistance circuit-based training in hypoxia on body composition and strength performance. *Eur. J. Sport Sci.* 19 1–11. 10.1080/17461391.2018.1564796 30638154

[B34] McMillan L. B. Zengin A. Ebeling P. R. Scott D. (2017). Prescribing physical activity for the prevention and treatment of osteoporosis in older adults. *Healthcare* 5:E85. 10.3390/healthcare5040085 29113119 PMC5746719

[B35] Mikhael M. Orr R. Fiatarone Singh M. A. (2010). The effect of whole body vibration exposure on muscle or bone morphology and function in older adults: a systematic review of the literature. *Maturitas* 66 150–157. 10.1016/j.maturitas.2010.01.013 20171817

[B36] Millet G. P. Debevec T. Brocherie F. Malatesta D. Girard O. (2016). Therapeutic use of exercising in hypoxia: promises and limitations. *Front. Physiol.* 7:224 10.3389/fphys.2016.00224 PMC4902009 27375500

[B37] Moreira-Marconi E. Dionello C. F. Morel D. S. Sa-Caputo D. C. Souza-Goncalves C. R. Paineiras-Domingos L. L. (2016). Could whole body vibration exercises influence the risk factors for fractures in women with osteoporosis? *Osteoporos. Sarcopenia* 2 214–220. 10.1016/j.afos.2016.09.003 30775489 PMC6372741

[B38] Navarrete-Opazo A. Mitchell G. S. (2014). Therapeutic potential of intermittent hypoxia: a matter of dose. *Am. J. Physiol. Regul. Integr. Comp. Physiol.* 307 R1181–R1197. 10.1152/ajpregu.00208.2014 25231353 PMC4315448

[B39] O’Brien K. A. Pollock R. D. Stroud M. Lambert R. J. Kumar A. Atkinson R. A. (2018). Human physiological and metabolic responses to an attempted winter crossing of Antarctica: the effects of prolonged hypobaric hypoxia. *Physiol. Rep.* 6:e13613. 10.14814/phy2.13613 29521037 PMC5843758

[B40] Ramos Campo D. J. Rubio Arias J. A. Jimenez Diaz J. F. (2015). Effects in body composition and bone mineral density of simulate altitude program in triathletes. *Nutr. Hosp.* 32 1252–1260. 10.3305/nh.2015.32.3.9386 26319847

[B41] Reginster J. Y. Burlet N. (2006). Osteoporosis: a still increasing prevalence. *Bone* 38(2 Suppl. 1), S4–S9. 10.1016/j.bone.2005.11.024 16455317

[B42] Rhea M. R. (2004). Determining the magnitude of treatment effects in strength training research through the use of the effect size. *J. Strength Cond. Res.* 18 918–920. 10.1519/14403.1 15574101

[B43] Ribeiro A. C. Sávio K. E. O. Rodrigues M. L. C. F. Costa T. H. M. Schmitz B. A. S. (2006). Validation of a food frequency questionnaire for the adult population. *Rev. Nutr.* 19 553–562.

[B44] Rittweger J. Debevec T. Frings-Meuthen P. Lau P. Mittag U. Ganse B. (2016). On the combined effects of normobaric hypoxia and bed rest upon bone and mineral metabolism: Results from the PlanHab study. *Bone* 91 130–138. 10.1016/j.bone.2016.07.013 27443510

[B45] Rosenberg D. E. Lee A. K. Anderson M. Renz A. Matson T. E. Kerr J. (2018). Reducing sedentary time for obese older adults: protocol for a randomized controlled trial. *JMIR Res Protoc.* 7:e23. 10.2196/resprot.8883 29434012 PMC5826980

[B46] Sallis R. (2015). Exercise is medicine: a call to action for physicians to assess and prescribe exercise. *Phys. Sportsmed* 43 22–26. 10.1080/00913847.2015.1001938 25684558

[B47] Schega L. Peter B. Torpel A. Mutschler H. Isermann B. Hamacher D. (2013). Effects of intermittent hypoxia on cognitive performance and quality of life in elderly adults: a pilot study. *Gerontology* 59 316–323. 10.1159/000350927 23652274

[B48] Schutzer K. A. Graves B. S. (2004). Barriers and motivations to exercise in older adults. *Prev. Med.* 39 1056–1061. 10.1016/j.ypmed.2004.04.003 15475041

[B49] Scott B. R. Slattery K. M. Sculley D. V. Dascombe B. J. (2014). Hypoxia and resistance exercise: a comparison of localized and systemic methods. *Sports Med.* 44 1037–1054. 10.1007/s40279-014-0177-177 24715613

[B50] Semenza G. L. (2001). HIF-1 and mechanisms of hypoxia sensing. *Curr. Opin. Cell Biol.* 13 167–171. 10.1016/s0955-0674(00)00194-0 11248550

[B51] Semenza G. L. (2012). Hypoxia-inducible factors in physiology and medicine. *Cell* 148 399–408. 10.1016/j.cell.2012.01.021 22304911 PMC3437543

[B52] Serebrovskaya T. V. Nikolsky I. S. Nikolska V. V. Mallet R. T. Ishchuk V. A. (2011). Intermittent hypoxia mobilizes hematopoietic progenitors and augments cellular and humoral elements of innate immunity in adult men. *High Alt. Med. Biol.* 12 243–252. 10.1089/ham.2010.1086 21962068 PMC3186684

[B53] Stadelmann K. Latshang T. D. Lo Cascio C. M. Clark R. A. Huber R. Kohler M. (2015). Impaired postural control in healthy men at moderate altitude (1630 m and 2590 m): data from a randomized trial. *PLoS One* 10:e0116695. 10.1371/journal.pone.0116695 25723529 PMC4344242

[B54] Terzi R. Yilmaz Z. (2016). Bone mineral density and changes in bone metabolism in patients with obstructive sleep apnea syndrome. *J. Bone Miner. Metab.* 34 475–481. 10.1007/s00774-015-0691-1 26204846

[B55] Tomiyama H. Okazaki R. Inoue D. Ochiai H. Shiina K. Takata Y. (2008). Link between obstructive sleep apnea and increased bone resorption in men. *Osteoporos. Int.* 19 1185–1192. 10.1007/s00198-007-0556-0 18224268

[B56] Turner S. Torode M. Climstein M. Naughton G. Greene D. Baker M. K. (2011). A randomized controlled trial of whole body vibration exposure on markers of bone turnover in postmenopausal women. *J. Osteoporos.* 2011:710387. 10.4061/2011/710387 21772975 PMC3135216

[B57] Verschueren S. M. Bogaerts A. Delecluse C. Claessens A. L. Haentjens P. Vanderschueren D. (2011). The effects of whole-body vibration training and vitamin D supplementation on muscle strength, muscle mass, and bone density in institutionalized elderly women: a 6-month randomized, controlled trial. *J. Bone Miner. Res.* 26 42–49. 10.1002/jbmr.181 20648661

[B58] von Stengel S. Kemmler W. Engelke K. Kalender W. A. (2011). Effects of whole body vibration on bone mineral density and falls: results of the randomized controlled ELVIS study with postmenopausal women. *Osteoporos. Int.* 22 317–325. 10.1007/s00198-010-1215-1214 20306017

[B59] von Stengel S. Kemmler W. Engelke K. Kalender W. A. (2012). Effect of whole-body vibration on neuromuscular performance and body composition for females 65 years and older: a randomized-controlled trial. *Scand. J. Med. Sci. Sports* 22 119–127. 10.1111/j.1600-0838.2010.01126.x 20500555

[B60] Wang G. Wang J. Sun D. Xin J. Wang L. Huang D. (2016). Short-term hypoxia accelerates bone loss in ovariectomized rats by suppressing osteoblastogenesis but enhancing osteoclastogenesis. *Med. Sci. Monit.* 22 2962–2971. 10.12659/msm.899485 27550548 PMC5006713

[B61] Wang J. Shang F. Mei Q. Wang J. Zhang R. Wang S. (2008). NO-donating genistein prodrug alleviates bone loss in ovariectomised rats. *Swiss Med. Wkly.* 138 602–607. 18941946 10.4414/smw.2008.11940

[B62] Weeks B. K. Beck B. R. (2008). The BPAQ: a bone-specific physical activity assessment instrument. *Osteoporos. Int.* 19 1567–1577. 10.1007/s00198-008-0606-602 18414964

[B63] Winklmayr M. Kluge C. Winklmayr W. Kuchenhoff H. Steiner M. Ritter M. (2015). Radon balneotherapy and physical activity for osteoporosis prevention: a randomized, placebo-controlled intervention study. *Radiat. Environ. Biophys.* 54 123–136. 10.1007/s00411-014-0568-z 25274266

[B64] Wu C. Giaccia A. J. Rankin E. B. (2014). Osteoblasts: a novel source of erythropoietin. *Curr. Osteoporos. Rep.* 12 428–432. 10.1007/s11914-014-0236-x 25204993 PMC4349388

[B65] Wu C. Rankin E. B. Castellini L. Alcudia J. F. LaGory E. L. Andersen R. (2015). Oxygen-sensing PHDs regulate bone homeostasis through the modulation of osteoprotegerin. *Genes Dev.* 29 817–831. 10.1101/gad.255000.114 25846796 PMC4403258

